# Case Report: Hypertriglyceridemia and Premature Atherosclerosis in a Patient With Apolipoprotein E Gene ε*2*ε*1* Genotype

**DOI:** 10.3389/fcvm.2020.585779

**Published:** 2021-01-18

**Authors:** Alena S. Limonova, Alexandra I. Ershova, Alexey N. Meshkov, Anna V. Kiseleva, Mikhail G. Divashuk, Vladimir A. Kutsenko, Oxana M. Drapkina

**Affiliations:** ^1^Laboratory of Clinomics, National Medical Research Center for Therapy and Preventive Medicine of the Ministry of Healthcare of the Russian Federation, Moscow, Russia; ^2^Laboratory of Molecular Genetics, National Medical Research Center for Therapy and Preventive Medicine of the Ministry of Healthcare of the Russian Federation, Moscow, Russia; ^3^Kurchatov Genomics Center-All-Russia Research Institute of Agricultural Biotechnology, Moscow, Russia; ^4^Biostatistics Laboratory, National Medical Research Center for Therapy and Preventive Medicine of the Ministry of Healthcare of the Russian Federation, Moscow, Russia; ^5^Department of Theory of Probability, Department of Mechanics and Mathematics, Lomonosov Moscow State University, Moscow, Russia; ^6^National Medical Research Center for Therapy and Preventive Medicine of the Ministry of Healthcare of the Russian Federation, Moscow, Russia

**Keywords:** premature cardiovascular disease, triglyceride, genetic testing, familial dysbetalipoproteinemia, apolipoprotein E

## Abstract

We present a case of a 40-year-old male with premature atherosclerosis, with evidence of both eruptive and tendinous xanthomas, which could imply an increase in both low-density lipoprotein (LDL) and triglyceride (TG) levels. However, his LDL was 2.08 mmol/l, TG -11.8 mmol/l on rosuvastatin 20 mg. Genetic evaluation was performed using a custom panel consisting of 25 genes and 280 variants responsible for lipid metabolism. A rare ε*2*ε*1* genotype of apolipoprotein E was detected. The combination of clinical manifestations and genetic factors in this patient leads to the diagnosis of familial dysbetalipoproteinemia. Implementation of genetic testing into routine clinical practice could not only improve disease diagnostics and management, but also help prevent their development.

## Introduction

High level of triglycerides (TG) indicates an increase in the number of atherogenic lipoproteins. In different studies, various mechanisms of atherogenesis of triglyceride-rich lipoproteins (TRLs), which encompass chylomicrons, very low density lipoproteins and their remnants, were proposed and proved. Both clinical and epidemiological data and data from genetic studies (Mendelian randomization and genome-wide association studies) support the independent causal role of TRLs for atherosclerotic cardiovascular disease (CVD) (1).

Elevated blood TG level may be caused by primary or secondary factors, or a combination of both (2). Secondary factors include dietary causes (alcohol abuse, excess carbohydrates and fats), certain diseases (metabolic syndrome, hypothyroidism, and others), and some medications. Genetic causes include mono- and polygenic disorders. Monogenic lipid disturbances result from defects in genes encoding various apolipoproteins (*APOE, APOA5, APOC2, APOB*), lipoprotein lipase (*LPL*), lipase maturation factor 1 (*LMF1*), glycosylphosphatidylinositol-anchored high density lipoprotein binding protein 1 (*GPIHBP1*), and others (3).

Here, we present a case of a 40-year-old male with early atherosclerosis due to severe hypertriglyceridemia which resulted from a combination of *APOE* gene ε*2*ε*1* rare genotype and additional metabolic risk factors.

## Case Description

A 40-year-old Caucasian male was hospitalized with an acute coronary syndrome. For ~2 years before admission, the patient had transient tightness in the chest with subsequent gradual deterioration of exercise capacity. Three months ago, he experienced an abrupt exacerbation of his angina with frequent episodes of crushing chest pain accompanied by sweating, which arose during minimal physical activity, but he refused from hospitalization then. He was prescribed with rosuvastatin 20 mg, acetylsalicylic acid 100 mg, metoprolol 25 mg b.i.d, and isosorbide dinitrate 40 mg. During 2 weeks prior to his admission, he was unable to perform any daily activity without chest pain despite taking the prescribed medicine, and therefore, he was admitted to the hospital.

The patient was a smoker with an estimated 25 pack year smoking history. He did not measure his blood pressure (BP) regularly and his maximum level of BP was 160/90 mmHg. Other cardiovascular risk factors included obesity (body mass index of 32 kg/m^2^, abdominal circumference of 105 cm) and hyperlipidemia (total cholesterol of 13.7 mmol/l before statin therapy). The patient had never been diagnosed with pancreatitis. He also had a history of intermittent claudication. His family history was unremarkable. Evaluation of his only child (11-year old girl) is planned. Dietary history was noteworthy for excess carbohydrates, saturated and trans fats together with alcohol abuse (up to eight liters of beer per week).

**Figure 1:**
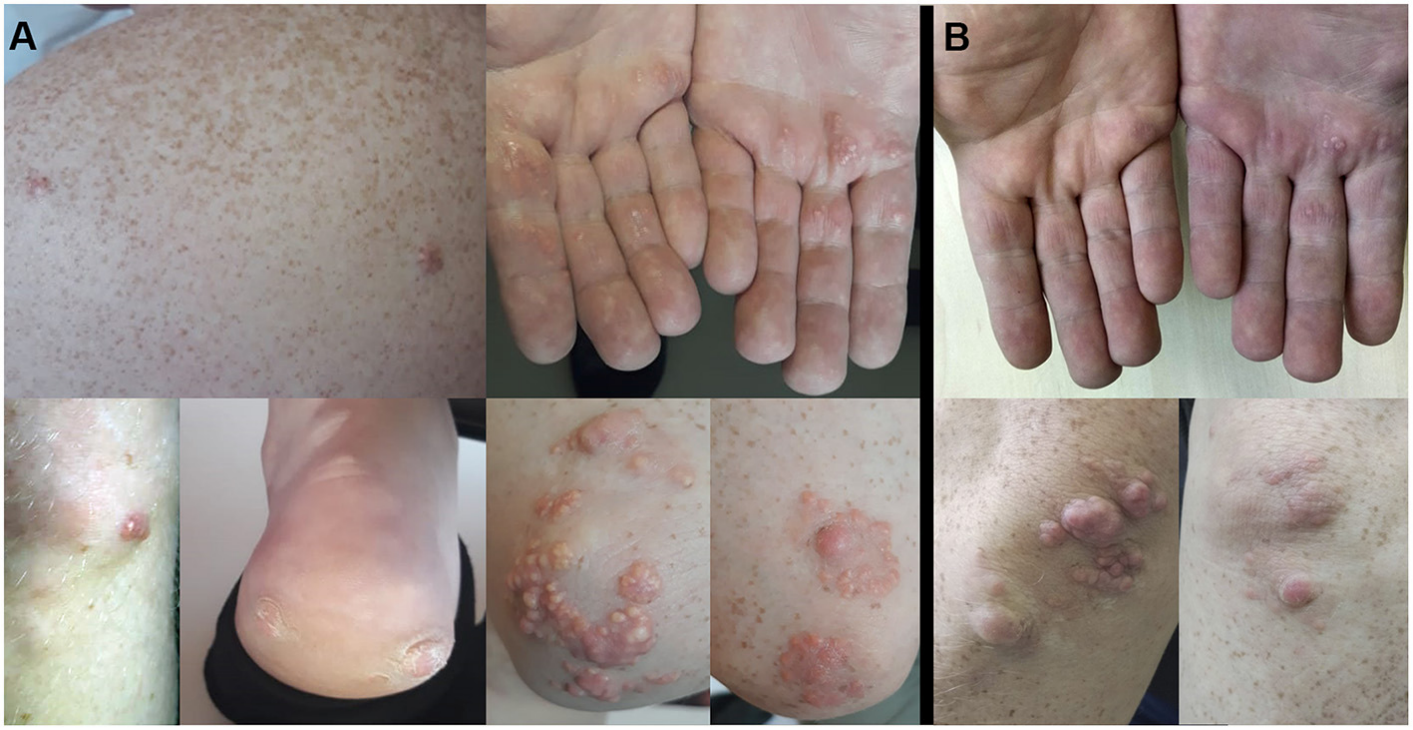
Eruptive xantomas on back, palms, knee, heel, elbows, before **(A)** and after treatment **(B)**.

Physical examination revealed multiple extensive tendinous xanthomas of Achilles tendons and flexor tendons of fingers, eruptive xanthomas of knees, elbows, back, palms and heels, as well as corneal arcus (see Figure 1A). Biochemical parameters are presented in Table 1. Lipoprotein profile assessment (Quantimetrix Lipoprint™ System) detected an increased amount of atherogenic lipoproteins [Very-low-density lipoprotein (VLDL) cholesterol subfraction of 173 mg/dl and presence of small dense LDL (LDL3 subfraction)]. Objective (abdominal obesity, elevated BP levels) and laboratory data [hypertriglyceridemia, high fasting glucose, low high-density lipoprotein cholesterol (HDL-C)] cluster in a metabolic syndrome.

**Table 1:** Laboratory data.

**Laboratory testing, units**	**On admission rosuvastatin 20 mg**	**2 days after admission rosuvastatin 40 mg**	**After 6 months of rosuvastatin 20 mg + fenofibrate 145 mg**
Total cholesterol, mmol/l	11.80	10.50	11.3
HDL-C, mmol/l	0.75	0.71	0.56
LDL-C, mmol/l		2.08	1.85
VLDL, mmol/l		3.87	
Triglycerides, mmol/l	12.45	8.43	12.68
Non–HDL-C, mmol/l		9.8	
Lipoprotein(a), mg/dL		24.3	
Alanine transaminase, IU/L	58		
Aspartate transaminase, IU/L	27		
Gamma-glutamyltransferase, IU/L	140		
Glucose, mmol/l	6.5		

**Figure 2:**
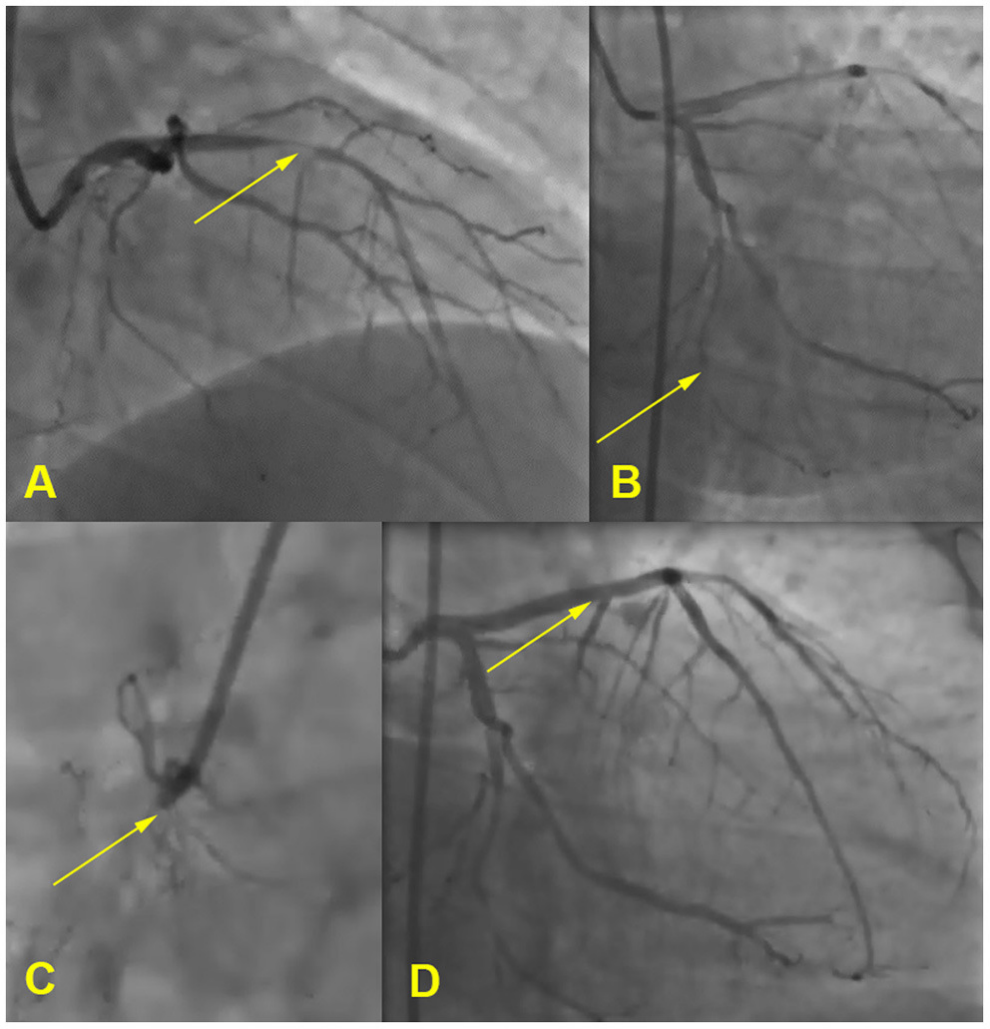
Coronary angiogram. **(A)** 80% occlusion of the proximal anterior descending artery, **(B)** 85% occlusion of the distal circumflex artery, **(C)** 100% occlusion of the proximal right coronary artery, **(D)** stent in anterior descending artery.

Electrocardiography showed sinus rhythm with Q-waves in leads III, aVF. A transthoratic echocardiogram was notable for an akinesis of basal and middle segments of inferior wall and a mild hypertrophy of the left ventricle. Coronary arteriography demonstrated the three-vessel disease: 80% occlusion of the proximal anterior descending artery, 85% occlusion of the distal circumflex artery, and 100% occlusion of the proximal right coronary artery. He received a drug eluting stent in anterior descending artery (see Figure 2). Duplex sonography revealed 40% stenosis of both femoral arteries, 50% stenosis of both superficial femoral arteries, and stenosis of carotid bifurcations and proximal internal carotid arteries up to 40% on the right and 50–55% on the left.

DNA extraction was performed using the QIAamp DNA Blood Mini Kit (Qiagen, Germany). Fluorometer Qubit 4.0 (Thermo Fisher Scientific, USA) was used for DNA quantification. Next-generation sequencing (NGS) was performed using the Ion S5 (Thermo Fisher Scientific, USA). Ampliseq libraries were prepared on the Ion Chef System (Thermo Fisher Scientific, USA) using the custom panel, created in the Ion AmpliSeq Designer (Thermo Fisher Scientific, USA). This panel consisted of 25 genes (*ABCA1, ABCG5, ABCG8, ANGPTL3, APOA1, APOA5, APOB, APOC2, APOC3, APOE, CETP, GPD1, GPIHBP1, LCAT, LDLR, LDLRAP1, LIPC, LIPI, LMF1, LPL, MTTP, PCSK9, SAR1B, STAP1, USF1*) and 280 variants responsible for lipid metabolism. After a bioinformatics analysis, .bam and .vcf files were obtained. For clinical interpretation only variants with frequencies <0.5% in the database gnomAD were further analyzed according to the ACMG/AMP2015 recommendations.

This patient had a rare ε*2*ε*1* genotype which was defined by the following variants: heterozygous pathogenic variant rs267606664 [NP_000032.1: p.G145D (p.G127D)] and homozygous pathogenic variant rs7412 [NP_000032.1: p.R176C (p.R158C)]. These variants were confirmed by Sanger sequencing on the 3500 DNA Analyzer (Thermo Fisher Scientific, USA). All steps were fulfilled according to the manufacturer's protocols. According to literature, in ε*2*ε*2* subjects some additional genetic factors may contribute to hypertriglyceridemia development. Henneman et al. investigated the variants of *APOC3, APOA5*, hepatic lipase, and *LPL* genes in two groups of homozygous ε*2*ε*2* individuals with normal or increased TG levels (4). It was shown that the frequency of *APOC3* rare variant (rs5128, 3238 G > C) was significantly higher in type III hyperlipoproteinemia. Our patient was found to be CC homozygous for this variant. Another variant of *APOA5* (rs651821), identified in this patient, is significantly more prevalent in subjects with severe hypertriglyceridemia compared with controls (5).

Score scales (6–8) were used for the assessment of the patient's risk for polygenic hypertriglyceridemia. Based on these scales, a risk score was calculated for each of the 1,786 individuals sampled from the generic Russian population (9) and the subject patient. The subject patient's risk score lay within the 2σ interval for each of the scores. These results support the absence of polygenic hypertriglyceridemia in this patient.

The patient was discharged from the hospital with prescriptions of rosuvastatin 40 mg, fenofibrate 145 mg, bisoprolol 5 mg, enalapril 5 mg b.i.d., and dual antiplatelet therapy. After 6 months of fenofibrate therapy, during a follow-up visit, it was noted that the skin xantomas became less prominent (see Figure 1B). However, the patient's adherence to recommendations about lifestyle modifications was poor and he lowered the prescribed statin dose himself.

## Discussion

APOE plays the most important role for mediating LDL receptor (LDLR)-dependent clearance of TRLs remnants from the circulation into the liver, as it is the ligand for the LDLR family of proteins and heparan sulfate proteoglycans (HSPG). In humans, there are three main isoforms of APOE protein (APOE2, APOE3, and APOE4) which arise from three allelic variants [ε*2*, ε*3*, and ε*4*, overall frequencies are 0.07, 0.82, and 0.11, respectively (10)]. These allelic variants are defined by two exonic *APOE* variants (p.C112R, p.R176C) (11).

The association of *APOE* variants with the lipid profile and CVD is widely investigated. Higher TG levels are associated with ε*2* allelic variant (10, 12). Recent meta-analyses investigating the significance of *APOE* variant on the development of CVD have led to controversial results. According to one of them, no significant association between ε*2* or ε*3* and the susceptibility to atherosclerosis was demonstrated, while ε*4* could be associated with clinical atherosclerosis (13). According to another one, ε*2 APOE* allele may appear as a risk factor for premature CVD in Asians while has a protective role in Caucasians as well as ε*4* allele acts as a genetic risk factor for premature CVD (14). No association of ε*2* allele with the risk of CVD was demonstrated when comparing groups with and without CVD (15). In healthy men, ε*2* allele was associated with a smaller LDL size which are known to be more atherogenic than larger ones (16). Within patients with CVD, carriers of ε*2* had smaller LDL levels when compared to non-carriers (17). A recent study showed an association between *APOE* variants and high-risk CVD marker ceramides in the blood (18).

Among homozygous ε*2*ε*2* individuals, only a few develop familial dysbetalipoproteinemia, a condition which is characterized by a combination of ε*2*ε*2* genotype and accumulation of the TRLs. As ε*2*ε*2* genotype is not enough for clinical manifestation, additional factors are needed like obesity, insulin resistance, type 2 diabetes mellitus, alcohol consumption, pregnancy, estrogen depletion, intake of certain medications, polymorphisms of lipolysis genes, advanced age, or menopause. Effect of these additional factors is mediated by increased VLDL production, decreased TRLs remnants' clearance, or TG lipolysis. The APOE2 protein, resulting from ε*2*ε*2* genotype, has a lower binding affinity to LDLR compared to other isoforms (APOE3 and APOE4) (19). Amino acid substitution at position 176 in APOE2 disrupts its binding to LDLR, but not to HSPG. The latter has a large clearance capacity and prevents remnant accumulation even in case of no LDLR being present. This mechanism partially explains why ε*2*ε*2* genotype alone does not necessary lead to familial dysbetalipoproteinemia (11). But HSPG mediated uptake in the liver of TRLs is substantially impaired in case of insulin resistance, when HSPG degradation occurs (19). This may be a link between metabolic disturbances and clinical manifestation of ε*2*ε*2* genotype.

One of the pathogenic variants found in this patient (p.G127D) has the highest population allele frequency of 0.0001 (4/31292) according to the gnomAD genome database, dataset version 2.1.1. It was first described in 1984 in a Finnish hypertriglyceridemic patient (20) with ε*3*ε*1* genotype. In this study on cultured fibroblasts, it was shown that this genotype led to reduced binding capacity to LDLR similar to that of APOE2. In another study, a case of hypertriglyceridemic patient homozygous for ε*1* was described as having lipid profile changes identical to those seen in patients homozygous for the ε*2* allele (21). A family of individuals with ε*2*ε*1* genotype was described in a previous study (22). But that family's TG level was in the range of 2.7–6.5 mmol/l, and neither of them had tendinous xanthomas or clinical manifestations of atherosclerosis. If more clinical examples of patients with rare genotypes were available, our understanding of their special characteristics would potentially be improved. Absence of polygenic hypertriglyceridemia in our patient, based on the risk scores calculated, may be interpreted as an emphasis on the importance of his *APOE* genotype for clinical manifestation. The limitation of the current work is no genetic and biochemical data being available from the relatives (with them residing too far away) which would have allowed to make a full characterization of genotype-phenotype correlations in this family pedigree.

Our patient had both clinical and laboratory manifestations (eruptive and tendinous xanthomas, elevation of TG, premature CVD), secondary risk factors (dietary factors, metabolic syndrome), and the appropriate genotype. All together, these indicators lead to the diagnosis of familial dysbetalipoproteinemia. This case of familial dysbetalipoproteinemia demonstrates prominent skin manifestations (combination of xantomas of all possible locations, including heels, which was not previously described in literature to our knowledge). Corneal arcus, observed in this patient, was found in a quarter of patients, referred to the lipid clinic due to high plasma TG level (23). It is of note, that in the same study only 8.5% patients had eruptive xantomas with TG levels exceeding 20.5 mM. Our patient exhibited both of these manifestations, though according to available medical documentation, his plasma TG level was in a range of 8–13 mM. In addition to an early onset of CVD (myocardial infarction with the three-vessel disease at the age of 40 years old), this patient had a pronounced atherosclerotic damage of other vascular regions (the brachiocephalic arteries and arteries of lower extremities). The increased risk for peripheral artery disease in ε*2*ε*2* individuals was previously described in literature (24). Atherosclerosis progression was most likely accelerated mainly by high TG levels, as the LDL level was initially (after correction for statin therapy) not so substantially elevated (about 4 mmol/l). It is assumed that defective binding of APOE2 to hepatic lipoprotein receptors, resulting in reduced hepatic cholesterol load, leads to the up-regulation of LDLR. Increased LDLR expression, along with lack of competition for binding by remnants of TRLs, results in low LDL level in ε*2*ε*2* individuals (25).

Genetic testing in this particular patient changed almost nothing for the management of his disease, but this is a vivid example of what could be prevented, if genetic testing were performed early in his childhood. It is of note, that childhood is a better period for instilling healthy lifestyle habits (26). Accordingly, primary prevention may be aimed at his only 11-year-old daughter. Reduced consumption of carbohydrates and fats, regular aerobic exercises, and weight control should be recommended. On the one hand, current guidelines for patients with dysbetalipoproteinemia are limited only to the control of secondary factors and use of either statin or fibrate therapy, or both (27, 28). Evidence for genetic testing of proband's relatives, their follow-up, and its cost-effectiveness is lacking. In a recent review, authors say that due to variable penetrance, cascade testing is now not recommended for familial dysbetalipoproteinemia (29). But on the other hand, existence of preventive options, based on genetic testing, is a point to be considered for prioritizing genetic tests (30). Moreover, non-clinical benefits of genetic testing should also be taken into account. Knowledge of the diagnosis, even when no interventional or preventive strategies are available, can enable the patients to get a better mental and emotional control over their health and health care. Such non-clinical consequences of genetic testing are labeled as patient empowerment (31). How genetic testing influences the individual's adherence to the given recommendations has been investigated among familial hypercholesterolemia patients (32, 33), but no evidence is available for familial dysbetalipoproteinemia. It is of note, that women have another important risk factor for developing hypertriglyceridemia, over and above, unhealthy lifestyle habits. Clinical cases, when the ε*2*ε*2* genotype was first found in a pregnant woman hospitalized with acute pancreatitis, are described in literature (34). It is really important that a woman and her physician are aware of the risk of such life-threating complications.

In summary, we report a 40-year-old patient with familial dysbetalipoproteinemia and rare ε*2*ε*1* genotype with premature atherosclerotic damage being observed in all main vascular regions. This clinical case demonstrates how genetic investigation could contribute to developing individualized and genetic-driven recommendations on lifestyle modifications. Implementation of genetic testing into routine clinical practice could not only improve disease diagnostics and management, but also help prevent its development.

## Data Availability Statement

Data supporting the findings of this study are available from the corresponding author upon reasonable request.

## Ethics Statement

Written informed consent was obtained from the individual(s) for the publication of any potentially identifiable images or data included in this article.

## Author Contributions

AL was treating the patient, performing his follow-ups, and wrote this paper. AE was treating the patient, supervising all parts of this paper's preparation, and edited this paper. AK performed the genetic testing, wrote the genetic part of this paper, and edited this paper. MD performed the Sanger sequencing. VK calculated the genetic risks scores. AM supervised the genetic testing of the patient and provided valuable comments on this case. OD is the chief of the center who provided valuable comments on this case. All authors contributed to the article and approved the submitted version.

## Conflict of Interest

The authors declare that the research was conducted in the absence of any commercial or financial relationships that could be construed as a potential conflict of interest.
